# A chromosome-level genome assembly of the redfin culter (*Chanodichthys erythropterus*)

**DOI:** 10.1038/s41597-022-01648-0

**Published:** 2022-09-01

**Authors:** Shihu Zhao, Xiufeng Yang, Bo Pang, Lei Zhang, Qi Wang, Shangbin He, Huashan Dou, Honghai Zhang

**Affiliations:** 1 College of Life Sciences, Qufu Normal University, Qufu, 273165 Shandong China; 2 Hulunbuir Academy of Inland Lakes in Northern Cold & Arid Areas, Hulunbuir, 021000 Inner Mongolia China

**Keywords:** Genetics, Evolution

## Abstract

*Chanodichthys erythropterus* is a fierce carnivorous fish widely found in East Asian waters. It is not only a popular food fish in China, it is also a representative victim of overfishing. Genetic breeding programs launched to meet market demands urgently require high-quality genomes to facilitate genomic selection and genetic research. In this study, we constructed a chromosome-level reference genome of *C. erythropterus* by taking advantage of long-read single-molecule sequencing and *de novo* assembly by Oxford Nanopore Technology (ONT) and Hi-C. The 1.085 Gb *C. erythropterus* genome was assembled from 132 Gb of Nanopore sequence. The assembled genome represents 98.5% completeness (BUSCO) with a contig N50 length of 23.29 Mb. The contigs were clustered and ordered onto 24 chromosomes covering roughly 99.49% of the genome assembly with Hi-C data. Additionally, 33,041 (98.0%) genes were functionally annotated from a total of 33,706 predicted protein-coding sequences by combining transcriptome data from seven tissues. This high-quality assembled genome will be a precious resource for future molecular breeding and functional genomics research of *C. erythropterus*.

## Abstract (summary)

**Table:** 

Measurement(s)	whole genome sequencing
Technology Type(s)	Oxford Nanopore Sequencing
Sample Characteristic - Organism	Chanodichthys erythropterus

## Background & Summary

*Chanodichthys erythropterus* (Basilewsky, 1855), which belongs to the family Cyprinidae, is widely spread in East Asia, inhabiting lakes or slow-moving rivers with rich vegetation^1^. Its juvenile fish feed on zooplankton, such as copepods, while adults mainly feed on small fish, a small and fierce carnivorous fish^2^. The *C. erythropterus* is highly adaptable to its natural environment and is not obviously affected even when living in alkaline lakes like Hulun Lake^3,4^.

Due to its delicious and delicate flesh, the *C. erythropterus* is so popular with consumers in the market and has a high commercial value^5^. Over the last decade, interest in the aquaculture of *C. erythropterus* has increased to meet market demand as wild stock is under threat due to overfishing and water pollution. Whole-genome sequencing of a given species is an important and essential tool to address important questions in both biological research and aquaculture. Former research on *C. erythropterus* has mostly focused on reproduction, age and growth^6,7^, feeding habits^2^, muscle composition^8^, and population genetics^9^. To date, no genomic resources are available for *C. erythropterus*, however, severely hampering research into its phylogeny, evolution and biology. Both genomic data and resources can provide a basis for our subsequent studies on the species diversity and population dynamics of *C. erythropterus*, and can provide a solid support for the proposal of logical conservation measures.

In the current study, the chromosome-level genome of *Chanodichthys erythropterus* was constructed using Nanopore sequencing and Hi-C technology. We have obtained a scaffold N50 of 42.39 Mb for the final genome assembly, which is approximately 1,085.51 Mb. Using Hi-C data, we identified that 99.49% of the assembled bases were associated with the 24 chromosomes. A valued resource for the conservation and breeding management of *C. erythropterus*, this genome could serve as the genetic basis for future research into its evolution and biology.

## Methods

### Sampling and sequencing

The *C. erythropterus* sample that was obtained in the Hulun Lake (Inner Mongolia, China) was used for genome sequencing and assembly. The muscle tissue was stored at −80 °C and used for DNA extraction, genomic DNA sequencing, and Hi-C library construction. We used a standard SDS extraction method to obtain high-molecular weight DNA.

Following the manufacturer’s recommendations, sequencing libraries were generated using the Truseq Nano DNA HT Sample Preparation Kit (Illumina, USA) and an index code was added to attribute sequences to each sample. These libraries constructed above were sequenced by the Illumina NovaSeq 6000 platform and yielded 150 bp paired-end reads with an insert size of approximately 350 bp. We obtained 41 Gb of raw genomic data for *C. erythropterus* as a result of Illumina sequencing.

Sequencing was performed on flow cells on the PromethION sequencer according to the manufacturer’s instructions. The Nanopore technology yielded 132 Gb of high-quality data from the long-read library, which covered 117.86-fold of the genome assembly.

In order to obtain chromosome-level assembly of the genome, a high-throughput chromatin conformation capture (Hi-C) library was built for sequencing^10^. We built the Hi-C library, which used original samples as input. Following grinding with liquid nitrogen, crosslinking was carried out with a 4% formaldehyde solution under vacuum for 30 minutes at room temperature. Add 2.5 M glycine to quench the cross-linking reaction for 5 minutes. Nuclei were digested with 100 units of MboI, tagged with biotin-14-dCTP and subsequently ligated with T4 DNA Ligase. The following incubation overnight to reverse cross-linking, the ligated DNA was segments sheared into 200 to 600 bp fragments. Blunt-end repair and A-tailing of DNA fragments followed by purification through biotin-streptavidin-mediated pulldown. The Hi-C libraries were eventually quantified and sequenced on Illumina PE150.

RNA was also extracted from seven tissues of the *C. erythropterus*, including intestine, liver, muscle, spleen, heart, gallbladder and kidney, transcriptome sequencing was performed on the Illumina NovaSeq 6000 platform and the resulting reads were used for gene prediction.

### Genome size estimation and contig assembly

**Fig. 1:**
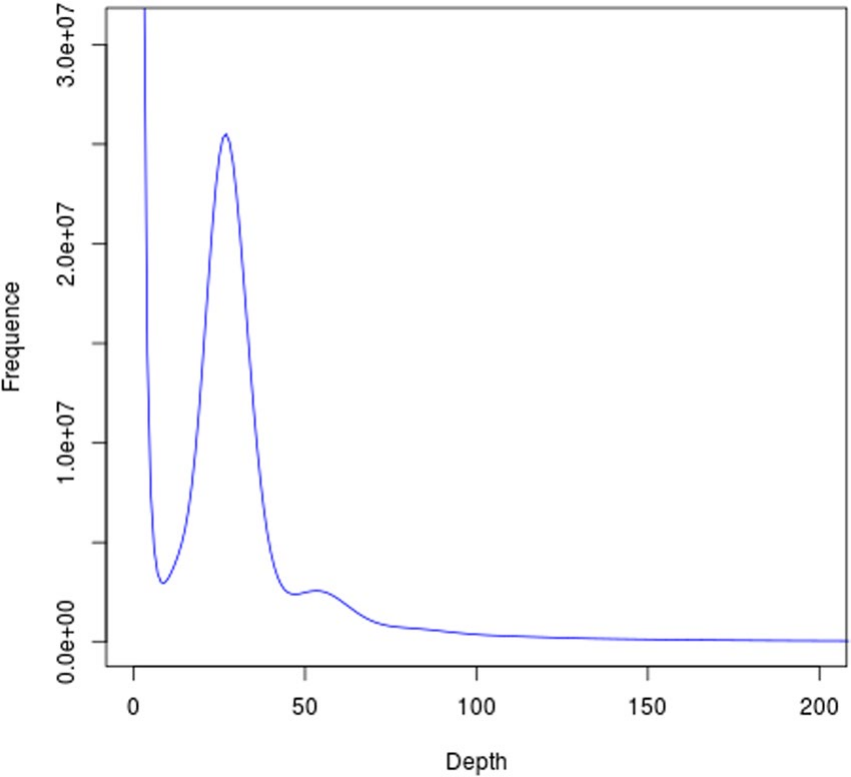
17-mer frequency distribution in *C. erythropterus* genome. The X-axis is the k-mer depth, and Y-axis represents the frequency of the k-mer for a given depth.

The Illumina data were analysed for k-mer depth frequency distribution to estimate the genome size, heterozygosity and the amount of repetitive sequences in *C. erythropterus*. The genome size (G) was estimated according to the following formula: G = k-mer number/k-mer depth, in which the k-mer number and k-mer depth are the total number and average depth of the 17 mers, respectively^11^. Using 41 Gb of clean Illumina data, the k-mer depth frequency distribution analysis was used for the genome of *C. erythropterus* (Fig. 1). On the basis of a total of 30,891,679,507 17-mer and a peak 17-mer depth of 27, the estimated genome size was 1120.68 Mb, the heterozygosity was 0.31%, and the amounts of repetitive sequences and guanine-cytosine were roughly 57.05% and 37.95%, respectively (Table 1).

**Table 1:** The result of k-mer analysis.

Kmer	Depth	N Kmer	Genomesize (M)	Heterozygousrate (%)	Repeatrate (%)
17	27	30,891,679,507	1,120.68	0.31	57.05

Using all Nanopore sequencing data, a preliminary assembly of the *C. erythropterus* genome was performed using NextDenovo assembler (v2.3.1) (https://github.com/Nextomics/NextDenovo) with the following parameters: “read_ cutoff = 1k, pa_correction = 20, sort_options = -m 20 g -t 10, correction_options = -p 10”. Finally, the contigs sequences were corrected by NextPolish (v1.3.1)^12^ using Illumina raw data as well as Nanopore sequencing data. Assembly of these data was then performed with NextDenovo, yielding a genome assembly of 1,085.49 Mb with a contig N50 of 23.28 Mb (Table 2). For this assembly, the length is the same as the genome size estimated by k-mer analysis.

**Table 2:** Assembly statistics of *C. erythropterus*.

Type	Contig length (bp)	Scaffold length (bp)	Contig number	Scaffold number
Total	1,085,492,200	1,085,510,300	231	50
Max	46,701,910	73,070,995	—	—
Number > = 2000	—	—	231	50
N50	23,286,394	42,399,299	18	11
N60	20,193,970	41,239,264	23	13
N70	13,953,221	39,512,133	29	16
N80	8,516,902	39,089,359	39	19
N90	3,227,172	37,095,974	60	21

### Chromosomal-level genome assembly using Hi-C data

**Fig. 2:**
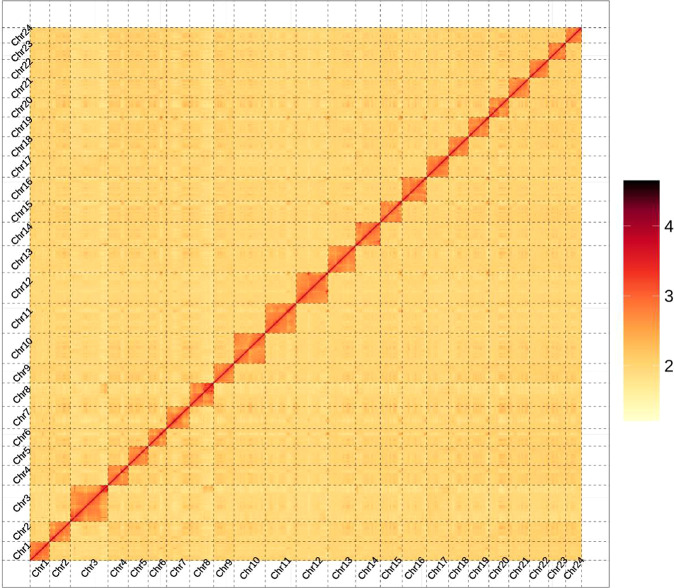
Hi-C chromosome contact map.

Through the use of the Hi-C scaffolding method^13^, the contigs in the initial assembly are anchored and oriented to the chromosomal scale of the assembly. The Hi-C library generated 86 Gb clean data. After the Hi-C corrected contigs were placed in the ALLhic pipeline^14^ for segmentation, orientation and sequencing, the final 99.49% of the assembled sequences were anchored to 24 pseudochromosomes with chromosome lengths that ranged from 31.72 Mb to 73.07 Mb (Table 3). This result is in agreement with the karyotype results which are based on cytological observations^15^, as many cyprinid fish such as *Ctenopharyngodon idellus*^16^, *Ancherythroculter nigrocauda*^17^, *Hypophthalmichthys molitrix* and *Hypophthalmichthys nobilis*^18^ with chromosome numbers of 2n = 48. Further we manually curated the Hi-C scaffolding from the chromatin contact matrix in Juicebox (Fig. 2). The 24 pseudochromosomes are easily distinguishable on the basis of the heatmap, and the strength of the interaction signal around the diagonal is fairly strong, indicating the high quality of this genome assembly. Following Hi-C correction, the final assembled genome was 1,085.51 Mb while the scaffold N50 was 42.39 Mb (Table 2). The genome size of *C. erythropterus* was similar to those of some cyprinid fishes such as the *Ctenopharyngodon idellus* (1.07 Gb), *Megalobrama amblycephala* (1.09 Gb)^19^, *Culter alburnus* (1.02 Gb)^19^, and *Ancherythroculter nigrocauda* (1.04 Gb), but much lower than that of the *Cyprinus carpio* (1.69 Gb)^20^.

**Table 3:** Summary of assembled 24 chromosomes of *C. erythropterus*.

Sequeues ID	Sequeues Length	Sequeues ID	Sequeues Length
Chr1	38,364,365	Chr13	54,232,047
Chr2	41,374,698	Chr14	47,491,587
Chr3	73,070,995	Chr15	42,777,030
Chr4	39,512,133	Chr16	48,609,862
Chr5	39,089,359	Chr17	42,399,299
Chr6	35,868,044	Chr18	39,783,364
Chr7	45,130,715	Chr19	39,191,619
Chr8	47,279,267	Chr20	39,167,548
Chr9	39,627,888	Chr21	41,239,264
Chr10	61,666,924	Chr22	37,095,974
Chr11	59,924,899	Chr23	33,623,848
Chr12	61,677,361	Chr24	31,722,787
Place	1,079,920,877		
Unplace	5,589,423		
Total	1,085,510,300		
Percentage	99.49%

### Assessment of the genome assemblies

For evaluating the accuracy and completeness of the genome assembly, we first compared Illumina reads to the assembly of *C. erythropterus* with the BWA (v0.7.8)^21^ in which 98.71% of the reads were able to be mapped to contigs. Additionally, we have assessed the integrity of the genome assembly with Benchmarking Universal Single-Copy Orthologs (BUSCO v5.2.1)^22^ with the vertebrata_odb10 database and CEGMA (v2.5)^23^. The final results of both showed that the assembly contained 98.5% of complete genes and 0.4% of fragmentarily conserved single-copy orthologs (Table 4), as well as 97.98% of the 248 core eukaryotic genes. All in all, the results of these assessments indicate to us that the *C. erythropterus* genome assembly is complete and of high quality.

**Table 4:** Results of the BUSCO assessment of *C. erythropterus*.

Type	Number
Complete BUSCOs (C)	3,304 (98.5%)
Complete and single-copy BUSCOs (S)	3,275 (97.6%)
Complete and duplicated BUSCOs (D)	29 (0.9%)
Fragmented BUSCOs (F)	14 (0.4%)
Missing BUSCOs (M)	36 (1.1%)
Total BUSCO groups searched	3,354

### Repeat annotation

Aiming to annotate repetitive elements in the *C. erythropterus* genome, methods combining homologous comparison and ab initio prediction were used. For ab initio repeat annotation, in which a de novo repetitive element database is constructed using LTR_FINDER (v1.0.7)^24^, RepeatScout (v1.0.5)^25^ and RepeatModeler (v1.0.8)^26^, the RepeatMasker (v4.0.5)^26^ was used to annotate the repeat elements in the database. The RepeatMasker and RepeatProteinMask (v4.0.5) were then used for known repeat element types via a search of the Repbase database^27^. Furthermore, TRF (v4.07b)^28^ can be used to annotate the tandem repeat. Ultimately, we identified 557 Mb of repetitive sequences, accounting for 51.34% of the assembled genome. These figures are higher than in *Ctenopharyngodon idellus* genome (38.06%) and *Megalobrama amblycephala* genome (38.68%), but slightly lower than that in *Danio rerio* genome (52.2%). Within this, we identified 469 Mb of LTR which dominated the assembled genome (43.23%) (Table 5).

**Table 5:** Classification of repeat elements in *C. erythropterus* genome.

Type	Denovo + Repbase		TE Proteins		Combined TEs	
	Length (bp)	% in Genome	Length (bp)	% in Genome	Length (bp)	% in Genome
DNA	58,226,942	5.36	7,413,708	0.68	62,122,195	5.72
LINE	7,641,127	0.70	16,986,628	1.56	20,557,781	1.89
SINE	1,634,833	0.15	0	0	1,634,833	0.15
LTR	467,225,494	43.04	32,239,687	2.97	469,221,600	43.23
Unknown	21,969,188	2.02	0	0	21,969,188	2.02
Total	551,340,511	50.79	56,626,202	5.22	557,279,616	51.34

### Gene prediction and annotation

**Fig. 3:**
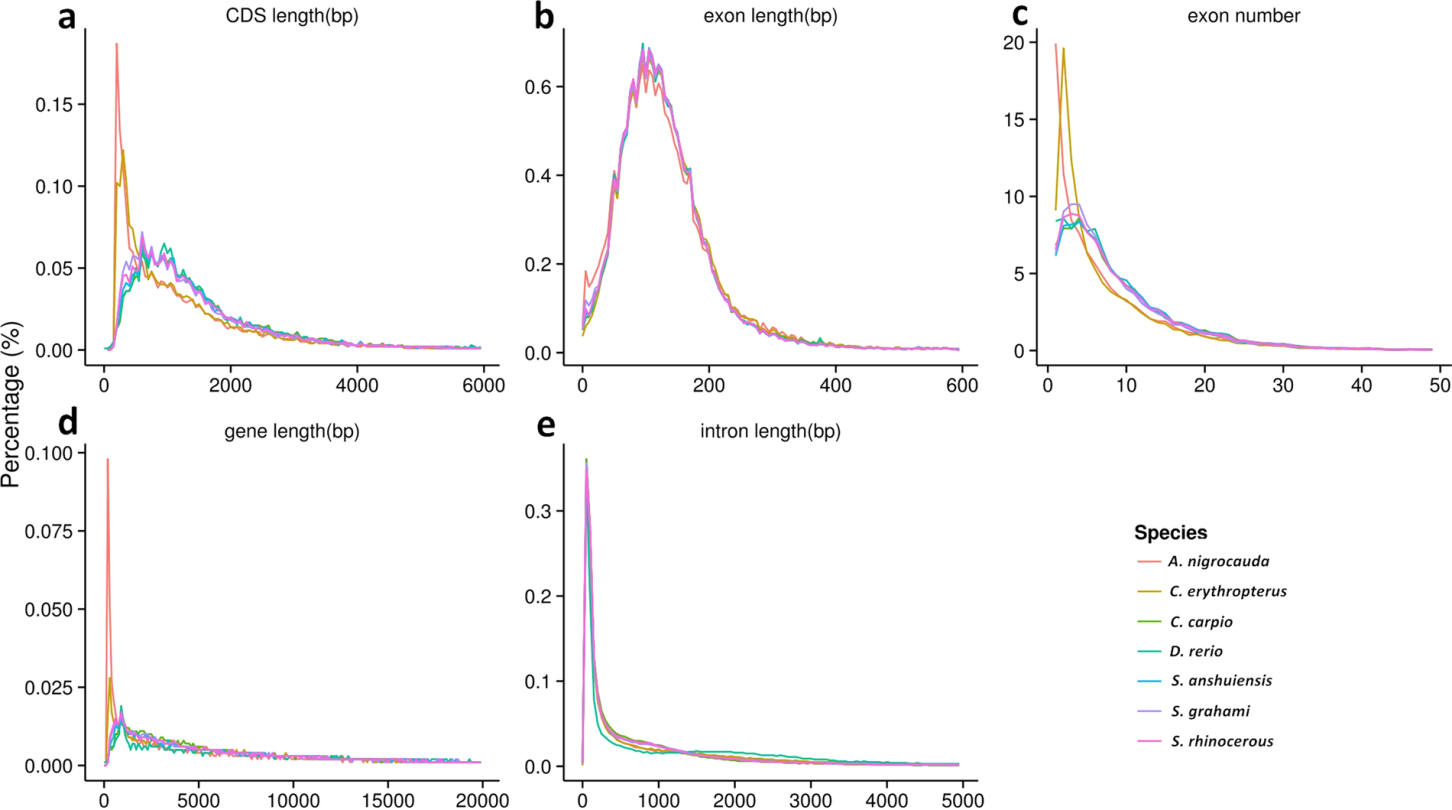
Comparisons of the prediction gene models in *C. erythropterus* genome to other species. (**a**) CDS length distribution and comparison with other species. (**b**) Exon length distribution and comparison with other species. (**c**) Exon number distribution and comparison with other species. (**d**) Gene length distribution and comparison with other species. (**e**) Intron length distribution and comparison with other species.

We detected protein-coding genes in the *C. erythropterus* genome assembly by a combination of three methods: Ab initio prediction, homology-based prediction and RNA-Seq prediction. As for ab initio prediction, Augustus (v3.2.3)^29^, GlimmerHMM (v3.04)^30^, SNAP (2013-11-29)^31^, Geneid (v1.4)^32^, and Genescan (v1.0)^33^ were used in our automated gene prediction pipeline. As for homology-based predictions, we downloaded the protein sequences of *Ancherythroculter nigrocauda* (GWHAAZV00000000), *Cyprinus carpio* (GCF_000951615.1), *Danio rerio* (GCF_000002035.6), *Sinocyclocheilus anshuiensis* (GCF_001515605.1), *Sinocyclocheilus grahami* (GCF_001515645.1), *Sinocyclocheilus rhinocerous* (GCF_001515625.1) from the NCBI database and used TblastN (v2.2.26)^34^ to match with the *C. erythropterus* genome with an e-value cutoff of 1E-5, and then the matched proteins were accurately spliced against the homologous genomic sequences using GeneWise (v2.4.1)^35^ software. As for RNA-Seq prediction, RNA-Seq data from seven tissues (including intestine, liver, muscle, spleen, heart, gallbladder and kidney) were aligned with genomic fasta using TopHat (v2.0.11)^36^ and gene structures were predicted using Cufflinks (v2.2.1)^37^. The non-redundant reference gene set was generated by combining genes predicted from three methods using EvidenceModeler (EVM, v1.1.1), using PASA (Program to Assemble Spliced Alignment) terminal exon support^38^, as well as including masked transposable elements as input to the gene predictions. Overall, a total of 33,706 protein-coding genes were predicted and annotated, with an average exon number per gene of 7.77 and an average CDS length of 1,363.50 bp (Table 6). In the final analysis, we compared the distribution of gene number, gene length, coding DNA sequence (CDS) length, exon length and intron length with that of other stiff bony fishes (Table 7 and Fig. 3).

**Table 6:** The statistics of gene models of protein-coding genes annotated in *C. erythropterus* genome.

Gene set		Number	Average transcript length (bp)	Average CDS length (bp)	Average exons per gene	Average exon length (bp)	Average intron length (bp)
*De novo*	Augustus	41,060	10,388.42	1,140.26	6.27	181.73	1,753.44
	GlimmerHMM	108,494	8,823.60	566.91	3.86	146.98	2,889.85
	SNAP	63,613	17,053.13	684.81	5.08	134.69	4,007.40
	Geneid	31,402	20,537.73	1,833.65	6.23	294.09	3,572.90
	Genscan	32,242	23,196.75	1,545.59	8.10	190.80	3,049.14
Homolog	*A. nigrocauda*	77,362	5,250.48	793.11	3.88	204.37	1,547.29
	*C. carpio*	32,561	11,939.92	1,570.24	6.95	225.83	1,741.90
	*D. rerio*	34,130	10,738.32	1,553.64	6.48	239.75	1,675.95
	*S. anshuiensis*	40,317	9,754.61	1,366.59	5.83	234.28	1,735.50
	*S. grahami*	41,063	8,962.70	1,270.36	5.57	228.06	1,683.09
	*S. rhinocerous*	34,358	11,162.86	1,430.97	6.45	222.02	1,787.22
RNAseq	PASA	116,439	12,899.85	1,279.78	7.79	164.34	1,711.96
	Cufflinks	80,918	18,982.81	3,213.28	8.52	376.93	2,095.63
EVM		37,168	14,243.82	1,274.10	7.17	177.66	2,101.51
PASA-update		36,819	14,260.02	1,288.94	7.22	178.52	2,085.34
Final set		33,706	15,469.83	1,363.50	7.77	175.58	2,085.05

**Table 7:** The comparison of the gene models annotated from *C. erythropterus* genome and other teleosts.

Species	Number	Average transcript length (bp)	Average CDS length (bp)	Average exons per gene	Average exon length (bp)	Average intron length (bp)
*C. erythropterus*	33,706	15,469.83	1,363.50	7.77	175.58	2,085.05
*S. anshuiensis*	42,645	17,491.76	1,690.94	9.95	169.90	1,765.00
*S. grahami*	45,899	16,217.28	1,585.31	9.23	171.79	1,778.31
*S. rhinocerous*	44,351	16,478.32	1,645.32	9.64	170.66	1,716.65
*A. nigrocauda*	34,414	15,105.52	1,309.42	7.86	166.68	2,012.35
*C. arpio*	43,518	15,745.34	1,727.67	9.94	173.73	1,567.13
*D. erio*	32,715	26,262.69	1,703.09	9.44	180.32	2,908.24

**Fig. 4:**
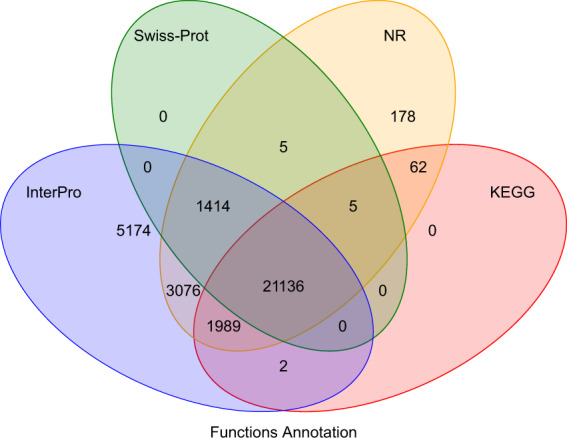
Venn diagram of the number of genes with functional annotation using multiple public databases.

The predicted genes of *C. erythropterus* were functionally annotated by using BLAST^39^ against SwissProt^40^, Nr from NCBI, KEGG^41^, InterPro^42^, GO^43^, and Pfam^44^ databases with an e-value cutoff of 1E-5. The InterproScan (v4.8)^45^ tool is used to predict protein function based on conserved protein structural domains using the InterPro database. The result was that 33,041 genes were successfully annotated for *C. erythropterus*, representing 98.0% of all predicted genes (Table 8 and Fig. 4).

**Table 8:** The number of genes with homology or functional classification for *C. erythropterus*.

Type	Number	Percent (%)
Total	33,706	—
SwissProt	22,560	66.9
Nr	27,865	82.7
KEGG	23,194	68.8
InterPro	32,791	97.3
GO	29,853	88.6
Pfam	21,159	62.8
Annotated	33,041	98.0
Unannotated	665	2.0

Eventually, miRNAs and snRNAs were identified via a search of the Rfam database using the default parameters of INFERNAL^46^. We chose the human rRNA sequences as a reference and used BLAST^39^ to predict the rRNA sequences of *C. erythropterus*. The tRNAs were predicted using the program tRNASCAN-SE^47^. As a result, we annotated 1,609 miRNA, 8,135 tRNA, 1,251 rRNA and 1,060 snRNA genes (Table 9).

**Table 9:** Classification of ncRNAs in *C. erythropterus* genome.

Type		Copy number	Average length (bp)	Total length (bp)	% of genome
miRNA		1,609	114.79	184,694	0.017014
tRNA		8,135	75.75	616,216	0.056767
rRNA	rRNA	1,251	133.09	166,498	0.015338
	18 S	49	448.49	21,976	0.002024
	28 S	105	278.25	29,216	0.002691
	5.8 S	8	157.00	1,256	0.000116
	5 S	1,089	104.73	114,050	0.010507
snRNA	snRNA	1,060	152.67	161,831	0.014908
	CD-box	231	145.46	33,601	0.003095
	HACA-box	93	151.15	14,057	0.001295
	splicing	690	155.31	107,164	0.009872

## Data Records

The genomic Illumina sequencing data were deposited in the Sequence Read Archive at NCBI SRR18691804^48^-SRR18691805^49^.

The genomic Nanopore sequencing data were deposited in the Sequence Read Archive at NCBI SRR18828942^50^.

The transcriptome Illumina sequencing data were deposited in the Sequence Read Archive at NCBI SRR18697292^51^-SRR18697298.

The Hi-C sequencing data were were deposited in the Sequence Read Archive at NCBI SRR18696935^52^.

The final chromosome assembly were deposited in the GenBank at NCBI JALPSW000000000^53^.

The annotation results of repeated sequences, gene structure and functional prediction were deposited in the Figshare database^54^.

## Technical Validation

The concentration of DNA was determined using Qubit Fluorometer and agarose gel electrophoresis, and the absorbance was approximately 1.8 at 260/280.

For the SNP discovery, Samtools (v0.1.19)^55^ was applied, resulting in the identification of 950,346 SNPs, including 947,721 heterozygous SNPs and 2,625 homozygous SNPs. The proportion of homozygous SNPs was extremely low, indicating the high accuracy of this assembly.

## Data Availability

No specific code or script was used in this work. The commands used in the processing were all executed according to the manuals and protocols of the corresponding bioinformatics software.
